# Phylogenetic diversity, antimicrobial susceptibility and virulence gene profiles of *Brachyspira hyodysenteriae* isolates from pigs in Germany

**DOI:** 10.1371/journal.pone.0190928

**Published:** 2018-01-11

**Authors:** Jessica Joerling, Stefanie A. Barth, Karen Schlez, Hermann Willems, Werner Herbst, Christa Ewers

**Affiliations:** 1 Institute of Hygiene and Infectious Diseases of Animals, Justus Liebig University Giessen, Giessen, Germany; 2 Friedrich-Loeffler-Institut/ Federal Research Institute for Animal Health, Institute of Molecular Pathogenesis, Jena, Germany; 3 Department of Veterinary Clinical Sciences, Clinic for Swine, Justus Liebig University Giessen, Giessen, Germany; Universidad Nacional de la Plata, ARGENTINA

## Abstract

Swine dysentery (SD) is an economically important diarrheal disease in pigs caused by different strongly hemolytic *Brachyspira* (*B*.) species, such as *B*. *hyodysenteriae*, *B*. *suanatina* and *B*. *hampsonii*. Possible associations of epidemiologic data, such as multilocus sequence types (STs) to virulence gene profiles and antimicrobial susceptibility are rather scarce, particularly for *B*. *hyodysenteriae* isolates from Germany. In this study, *B*. *hyodysenteriae* (n = 116) isolated from diarrheic pigs between 1990 and 2016 in Germany were investigated for their STs, susceptibility to the major drugs used for treatment of SD (tiamulin and valnemulin) and genes that were previously linked with virulence and encode for hemolysins (*tlyA*, *tlyB*, *tlyC*, *hlyA*, *BHWA1_RS02885*, *BHWA1_RS09085*, *BHWA1_RS04705*, and *BHWA1_RS02195*), outer membrane proteins (OMPs) (*bhlp16*, *bhlp17*.*6*, *bhlp29*.*7*, *bhmp39f*, and *bhmp39h*) as well as iron acquisition factors (*ftnA* and *bitC*). Multilocus sequence typing (MLST) revealed that 79.4% of the isolates belonged to only three STs, namely ST52 (41.4%), ST8 (12.1%), and ST112 (25.9%) which have been observed in other European countries before. Another 24 isolates belonged to twelve new STs (ST113-118, ST120-123, ST131, and ST193). The temporal distribution of STs revealed the presence of new STs as well as the regular presence of ST52 over three decades (1990s–2000s). The proportion of strains that showed resistance to both tiamulin und valnemulin (39.1%) varied considerably among the most frequent STs ranging from 0% (0/14 isolates resistant) in ST8 isolates to 46.7% (14/30), 52.1% (25/48), and 85.7% (6/7) in isolates belonging to ST112, ST52, and ST114, respectively. All hemolysin genes as well as the iron-related gene *ftnA* and the OMP gene *bhlp29*.*7* were regularly present in the isolates, while the OMP genes *bhlp17*.*6* and *bhmp39h* could not be detected. Sequence analysis of hemolysin genes of selected isolates revealed co-evolution of *tlyB*, *BHWA1_RS02885*, *BHWA1_RS09085*, and *BHWA1_RS02195* with the core genome and suggested independent evolution of *tlyA*, *tlyC*, and *hlyA*. Our data indicate that in Germany, swine dysentery might be caused by a limited number of *B*. *hyodysenteriae* clonal groups. Major STs (ST8, ST52, and ST112) are shared with other countries in Europe suggesting a possible role of the European intra-Community trade of pigs in the dissemination of certain clones. The identification of several novel STs, some of which are single or double locus variants of ST52, may on the other hand hint towards an ongoing diversification of the pathogen in the studied area. The linkage of pleuromutilin susceptibility and sequence type of an isolate might reflect a clonal expansion of the underlying resistance mechanism, namely mutations in the ribosomal RNA genes. A linkage between single virulence-associated genes (VAGs) or even VAG patterns and the phylogenetic background of the isolates could not be established, since almost all VAGs were regularly present in the isolates.

## Introduction

Swine dysentery (SD) belongs to one of the most challenging diarrheal infections in pigs worldwide with mortality up to 30% [1]. The disease is caused by the strongly hemolytic *Brachyspira* (*B*.) species *B*. *hyodysenteriae*, *B*. *suanatina* and *B*. *hampsonii* with *B*. *hyodysenteriae* being the most relevant species [2]. However, *B*. *hyodysenteriae* can also be isolated from apparently healthy pigs [3]. Hitherto, the novel strongly hemolytic *B*. *suanatina* has been only isolated from pigs in Scandinavia where this pathogen caused damages consistent with mild SD [2, 4]. Until now, *B*. *suanatina* has no clinical relevance in other pig-producing countries. In contrast, *B*. *hampsonii*, which was initially described in the United States of America and Canada [5], has already been detected in pigs from Europe, either during routine diagnostic screening or as part of monitoring programs, but without any linkage to SD so far [6, 7]. To differentiate these newly emerged strongly hemolytic species from *B*. *hyodysenteriae*, a PCR method was established since biochemical tests failed for specific species identification [8, 9].

Some of the major issues of research in the field of swine dysentery currently touch aspects of the population structure and diversity of *B*. *hyodysenteriae*, the presence and distribution of virulence-associated genes (VAGs), and the antimicrobial susceptibility of the pathogen to pleuromutilins [2]. Recent studies employing multilocus sequence typing (MLST) have identified different major clonal groups among *B*. *hyodysenteriae* isolates [10, 11, 12, 13, 14] that sometimes reflected the geographical origin of the isolates. Furthermore, combining MLST with data of antimicrobial susceptibility revealed the accumulation of pleuromutilin-susceptible isolates in specific clonal complexes [14, 15]. Antibiotics such as pleuromutilins, tylosin, and lincomycin are the drugs most commonly used for the treatment of SD [1]. However, due to their wide use increasing resistance of *B*. *hyodysenteriae* isolates to these substances has become a global matter of concern. The pleuromutilin antibiotics tiamulin and valnemulin target the peptidyl transferase center (PTC), including parts of the 23S rRNA and the ribosomal protein L3. Previous studies demonstrated a significant association between pleuromutilin susceptibility and a single nucleotide change in the ribosomal protein L3 gene at position 443 (amino acid change Asn148Ser) [16, 17].

*B*. *hyodysenteriae* isolates show differences in their virulence which may be due to the presence of certain VAGs [18, 19]. Genes encoding hemolysins, outer membrane proteins, iron uptake systems, flagellin or NADH-oxidase have been associated with virulence [20, 21, 22, 23]. A pathogenic relevance has also been suggested for six genes located on a recently identified 36 kb circular plasmid [3]. However, recent comparative studies including genomes of virulent and avirulent strains indicated that the relevance of the plasmid genes for virulence remains elusive [3]. So far, the presence of plasmid genes and other VAGs of *B*. *hyodysenteriae* could not unalterably be linked to the sequence type [3, 24].

The present study aimed to determine the clonal origin, pleuromutilin susceptibility, and virulence gene profile of *B*. *hyodysenteriae* isolates and to investigate possible associations between these features to broaden the knowledge about the epidemiology of this important swine pathogen.

## Materials and methods

### *Brachyspira* isolates and culture conditions

Strongly hemolytic *B*. *hyodysenteriae* isolates (n = 116) were isolated from fecal samples of diarrheic pigs from farms (n = 100) mainly located in Hesse and Northern Germany from 1990–2016. A variable number of isolates (2–11 samples) was repeatedly recovered from six pig farms (no. 31 (3 isolates), 40 (11), 43 (2), 50 (2), 81 (2), and 95 (2); S2 Table). Times between submissions varied between two weeks and 19 months. *B*. *hyodysenteriae* reference strains B204 [25] and B8044 [26], both isolated from pigs with swine dysentery in the United States, were also included in this study. Bacteria were grown on trypticase soy agar (TSA) plates containing 5% (v/v) sheep blood and different antibiotics (6.23 μg/ml colistin, 6 μg/ml vancomycin, 12 μg/ml spectinomycin, 15.25 μg/ml spiramycin and 12.5 μg/ml rifampicin) under anaerobic condition if not stated otherwise [27]. The purity of isolates was tested macroscopically and by using dark-field and phase-contrast microscopy (Leica DMR HC microscope, Leitz, Wetzlar, Germany). *B*. *hyodysenteriae* isolates were confirmed by PCR targeting the *nox*-gene as previously described [28].

### Multilocus sequence typing (MLST)

MLST was performed according to previously published protocols [10, 11] targeting genes that encode for the following enzymes: alcohol dehydrogenase (*adh*), alkaline phosphatase (*alp*), esterase (*est*), glutamate dehydrogenase (*gdh*), glucose kinase (*glpk*), phosphoglucomutase (*pgm*), and acetyl-CoA acetyltransferase (*thi*). PCR amplicons were sequenced by LGC Genomics (Berlin, Germany) and sequences were analyzed using Ridom seqsphere version 1.0.1 (http://www.ridom.de/seqsphere/). Isolates were assigned to sequence types (STs) according to the allele profiles provided on the *Brachyspira* MLST website (http://pubmlst.org/brachyspira/). New STs and alleles were submitted to the curator of the website for assignments. To put our isolates into a global and temporal context, we included 421 *B*. *hyodysenteriae*-isolates from the MLST website, 52 isolates previously published from Spain and Portugal [12] as well as 160 additional isolates from Italy [14]. Isolates that shared at least six of the seven loci have been defined as a clonal complex (CC). eBURST analysis (http://eburst.mlst.net/) has been employed to determine the putative founder of a clonal complex, which determines the CC number.

### Antimicrobial susceptibility testing and identification of single nucleotide polymorphisms in the ribosomal protein L3 gene

Antimicrobial susceptibility testing of *B*. *hyodysenteriae* isolates to tiamulin and valnemulin (Sandoz, Kundl, Austria) was performed as recently described [17]. Briefly, with 100 μl of the antimicrobial substances two-fold serial dilutions in BHI were prepared in micro titer plates (MTP; Greiner Bio-One, Frickenhausen, Germany). From each isolate, a bacterial suspension with an optical density of McFarland standard 1, which is equivalent to approximately 3 x 10^7^ CFU (Colony Forming Units) was prepared [29]. One milliliter was diluted in 14 ml Brain-Heart-Infusion broth (BHI; Oxoid, Wesel, Germany) supplemented with 20% fetal calf serum (FCS; Biochrom, Berlin, Germany), and 100 μl each were distributed on the wells of a 96-well MTP, resulting in 2 x 10^5^ CFU (absolute number) per well. MTPs were incubated under anaerobic conditions at 37°C on a shaker (Edmund Bühler, Hechingen, Germany) with 125 circular motions per minute. The last dilution that inhibited bacterial growth was recorded as the minimal inhibitory concentration (MIC). MIC_50_ and MIC_90_ are defined as those concentrations at which 50% and 90% of the isolates are inhibited in growth. MIC_90_ values were chosen to compare STs, whereas the MIC_50_ value was chosen to analyze the antimicrobial susceptibility of the isolates over time. Type strain B204 was used as an internal control on each MTP.

As approved standards for antimicrobial susceptibility testing of *B*. *hyodysenteriae* are not provided by the Clinical and Laboratory Standards Institute (CLSI) or by the European Committee on Antimicrobial Susceptibility Testing (EUCAST) MIC values were interpreted according to a previous publication [30] which was in line with the Swedish Veterinary Antimicrobial Resistance Monitoring program (SVARM).

To unveil pleuromutilin resistance mechanisms, i.e. previously reported single nucleotide polymorphisms inter alia in the ribosomal protein L3 gene (GenBank accession no. AF114845), we analyzed nucleotide sequences of the ribosomal protein L3 [17] from 31 isolates representing the most common STs of our study. For comparisons, the ribosomal protein L3 gene of the whole genome sequenced susceptible strain G21 (ST8 [31]) served as reference.

### Detection of virulence-associated genes (VAGs) by PCR

PCRs for the amplification of iron-related genes (*ftnA*, *bitC*) and genes encoding outer membrane proteins (*bhlp16*, *bhlp17*.*6*, *bhlp29*.*7*, *bhmp39f*, and *bhmp39h*) were performed according to published protocols [32, 33]. *Bhlp16* and *bhlp17*.*6* were formerly known as small membrane proteins (*smp*). Data were partly adopted from a recent study [32], while the number of isolates as well as the number of genes tested in the present study was significantly extended. Additionally, we tested all eight *B*. *hyodysenteriae* hemolysin genes *tlyA*, *tlyB*, *tlyC*, *hlyA*, *BHWA1_RS04705*, *BHWA1_RS09085*, *BHWA1_RS02195*, and *BHWA1_RS02885* [34, 35, 36, 37, 38]. One PCR reaction mix (30 μl total volume) contained 1 x NH_4_ buffer, 1 Unit Taq Polymerase (PANScript DNA-Polymerase, PAN Biotech, Aidenbach, Germany), 2 mM MgCl_2_, 133 μM of each nucleotide (Rapidozym, Berlin, Germany), 0.5 μM of each primer, and 3 μl template DNA. DNA was extracted from 200 μl BHI broth culture (Oxoid, Wesel, Germany) by use of guanidinium thiocyanate and diatomaceous earth (Sigma Aldrich Chemie, Munich, Germany) as described earlier [39]. Primers (Eurofins Genomics, Ebersberg, Germany), PCR conditions, and control strains are provided in S1 Table. Amplicons were separated by horizontal electrophoresis using 2% Tris-acetic acid-EDTA (TAE) agarose gels supplemented with 0.5 μg/ml ethidium bromide (Serva Electrophoresis, Heidelberg, Germany) and visualized by UV light.

### Determination of a possible link between the sequence type of *B*. *hyodysenteriae* and hemolysin gene sequences

For the concatenated sequences of the seven housekeeping genes and the sequences of seven hemolysin genes (excluding *BHWA1_RS04705*) of selected *B*. *hyodysenteriae* isolates distance matrices were created by use of the software Geneious version 8.1.8 (Biomatters, Auckland, New Zealand) based on the neighbour-joining-algorithm. The statistical calculation of the correlation between hemolysin genes and housekeeping genes was carried out with the Mantel test [40] in the open-source software R version 3.2.3. (https://www.r-project.org/) for the nucleotide sequences as well as for amino acid sequences. The nucleotide sequences of hemolysin genes have been deposited in GenBank under accession numbers MF488745—MF488776 (*hlyA*), MF488777—MF488802 (*tlyB*), MF488803—MF488834 (*BHWA1_RS02195*), MF488835—MF488864 (*tlyA*), MF488865—MF488893 (*tlyC*), MF488894—MF488925 (*BHWA1_RS09085*), and MF488926—MF488952 (*BHWA1_RSRS02885*).

### Statistics and data analysis

Data were analyzed using the software BioNumerics version 6.6 (Applied Maths, Sint-Martens-Latem, Belgium), SPSS Statistics version 22 (IBM, Armonk, United States) as well as Geneious version 8.1.8 (Biomatters, Auckland, New Zealand). The BURST algorithm of eBURST version 3 (http://eburst.mlst.net/) was used to determine the affiliation of isolates into CCs.

## Results

### MLST of *B*. *hyodysenteriae* isolates

**Fig 1:**
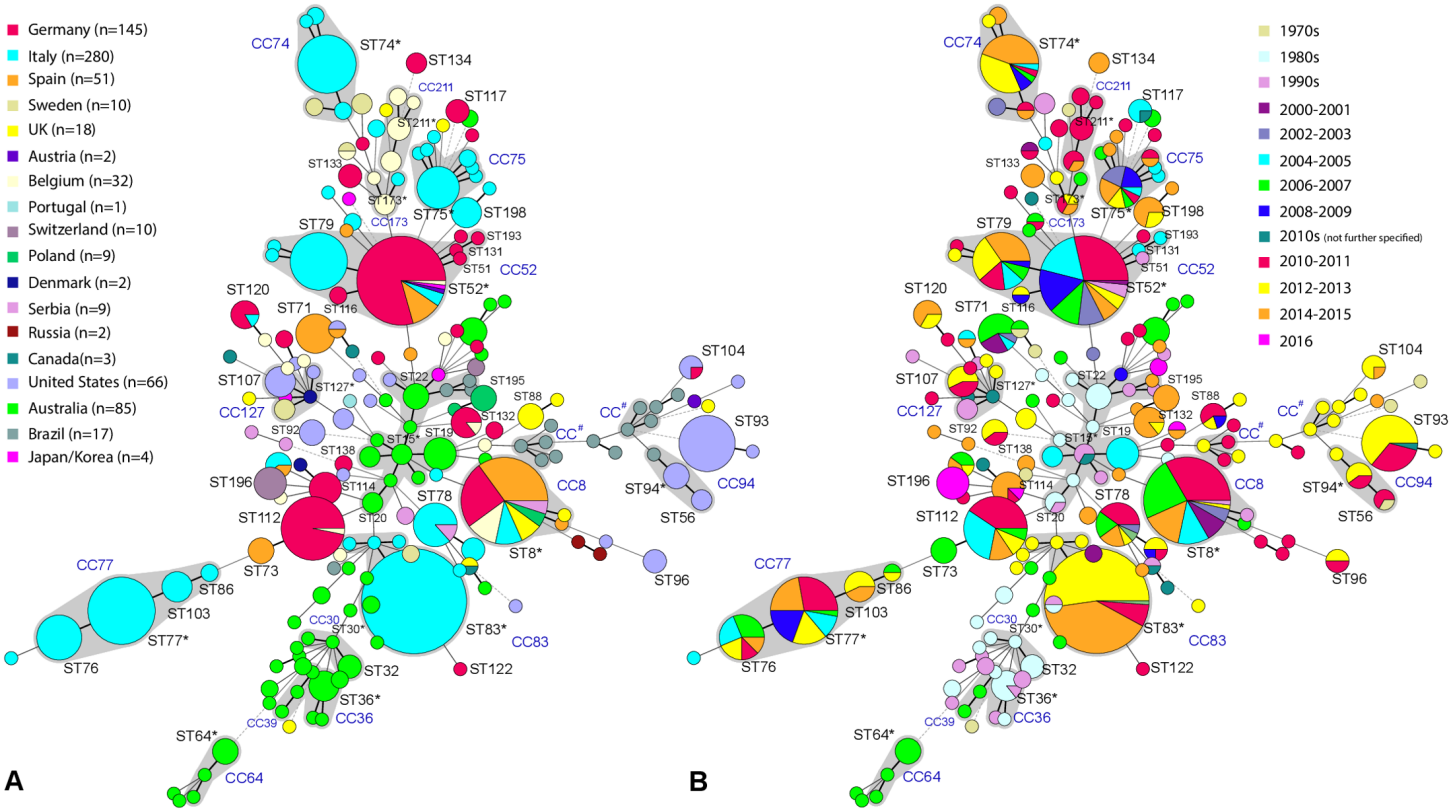
Fig 1A and 1B. MSTs of 746 *B*. *hyodysenteriae* isolates (n = 741 from pigs; n = 5 from birds and mice) from the MLST database and from this study. MST A mirrors the spatial distribution of isolates. Each circle represents a different sequence type (n = 186), the color indicates the country of origin and the size reflects the number of isolates deposited. MST B depicts the temporal distribution of isolates. Each circle represents a different year of isolation and the size reflects the number of isolates from the year in question. Clonal complexes (CC) of STs sharing at least six common loci are indicated by grey shading. The predicted founder of a CC is marked with an asterisk (*); # = CCs with unclear founders.

Among the 116 *B*. *hyodysenteriae* isolates, three STs, namely ST52 (41.4%), ST8 (12.1%), and the newly assigned ST112 (25.9%), occurred predominantly. The remaining isolates were distributed among twelve STs (ST113-118, ST120-123, ST131, and ST193), which were previously unknown and have been deposited into the MLST database. Based on the nucleotide allelic profiles of our *B*. *hyodysenteriae* isolates, 421 isolates deposited to the MLST database (http://pubmlst.org/brachyspira; last accessed 09/08/2017) and 212 isolates previously published but not submitted to the MLST database the eBURST analysis (S1 Fig) identified 17 CCs from the 186 STs of 746 *B*. *hyodysenteriae* isolates. The CCs are named after their proposed founder as indicated in both minimum spanning trees (MSTs) in Fig 1A and 1B. Novel STs from German isolates like ST116 or ST131 represent single locus variants (SLVs) of the already published ST52 (predicted founder of CC52), whereas ST193 is a double locus variant (DLV) of ST52. The new ST112 is a DLV of ST20, which was described from Australian isolates and differs in the *glpK* and *pgm* alleles.

The distribution of the 746 *B*. *hyodysenteriae* isolates is shown according to the country of origin (Fig 1A) and the year of isolation (Fig 1B) in the MSTs. Most (99.3%) of the 746 isolates originated from pigs, while few isolates were from birds (n = 3) and mice (n = 2). It becomes evident that the prominent STs identified in our study, namely ST8, ST52, and ST112, belong to separate clonal complexes. While the novel ST112 is limited to German and Belgian isolates only, the other two prominent STs also contain isolates from other European countries, i.e. Italy, Spain, Belgium, and Austria in the case of ST52, and Italy, the United Kingdom, Spain, Belgium, Poland, and Serbia in case of ST8. Except for ST120 that has been detected in Italy as well, the twelve novel STs appear unique to Germany. *B*. *hyodysenteriae* isolates from Europe, the United States of America, and from Australia hardly showed any overlap in their sequence types. Interestingly, ST104 contains isolates from Germany [3] and the United States of America. In addition, some countries, such as Italy, revealed the predominance of specific STs, such as ST75, ST77, and ST76 [15]. Regarding the temporal distribution of the 746 *B*. *hyodysenteriae* isolates the MST (Fig 1B) illustrates that a high number of isolates was obtained in the 2010s (n = 466) while from the 1990s, only eight isolates had been submitted to the MLST database. There are differences regarding the time of isolation among different STs. First, single STs or even CCs were only observed in the last century. This applies to isolates belonging to CC36 and CC30 which were only detected from the 1980s until the 1990s. Second, there are certain STs that were only obtained after the turn of century, like isolates of CC64 which were only detected in 2006/07. Furthermore, ST77, ST76, and ST83 were first detected in 2004. These three STs were continuously isolated until the end of our study period (2015/16). The novel sequence types ST94 and ST93 were only isolated between 2010 and 2013. Regarding the major STs in Germany, ST112 was not detected before the 2000s while the first ST8 and ST52 isolates were already present in the 1990s. While the detection rate of ST8 and ST52 isolates increased over time, the frequency of ST52 isolates peaked in the 2000s (35 isolates) and decreased in the 2010s (25 isolates).

Notably, the isolates recovered from repeated submissions in six pig farms (no. 31 (ST52), 40 (ST52), 43 (ST52), 50 (ST112), 81 (ST114), and 95 (ST120)) each belonged to the same ST (S2 Table).

### Antimicrobial susceptibility of *B*. *hyodysenteriae* and identification of single nucleotide polymorphisms in the ribosomal protein L3 gene

MIC values ranged from ≤0.016 μg/ml to >16 μg/ml for tiamulin (MIC_50_: 2 μg/ml) and ≤0.004 μg/ml to >4 μg/ml for valnemulin (MIC_50_: 0.5 μg/ml) (Table 1). Overall, 39.1% of our isolates were regarded resistant to both substances. An increase of pleuromutilin resistance was found in *B*. *hyodysenteriae* isolates recovered in intervals of three weeks and 19 months, respectively, from two of six pig farms (no. 43 and 95). No change in MIC values, however, was observed in the remaining isolates recovered from specimens submitted in intervals of two weeks up to 18 months (farms no. 31, 40, 50, 81) (S2 Table). The analysis of the mutation of the ribosomal protein L3 gene in 31 *B*. *hyodysenteriae* isolates of different STs at nucleotide position 443 (amino acid position 148), which has recently been linked with susceptibility to pleuromutilins [16, 17], revealed that all eight tested ST112 isolates lacked this mutation, but only four of them were resistant to at least one pleuromutilin (Table 2). Among seven L3 gene sequenced ST8 isolates, four isolates showed the specific Asn148Ser mutation, with three isolates being classified as susceptible to both pleuromutilins. Of 16 ST52 isolates only two revealed a non-synonymous mutation at the specific position in the L3 gene. Isolates of this ST that lacked the mutation were classified as intermediate or resistant.

**Table 1:** Minimum inhibitory concentration of pleuromutilins used in this study and classification of isolates as being resistant, intermediate or susceptible.

Pleuromutilin	Concentrations tested [μg/ml]	MIC [μg/ml]		
		Resistant	Intermediate	Susceptible
Tiamulin	0.016–16	>2	0.25–≤2	≤0.25
Valnemulin	0.004–4	>1	0.125–≤1	≤0.125

**Table 2:** Single nucleotide polymorphism in the *B*. *hyodysenteriae* ribosomal protein L3 gene/protein in relation to sequence type and pleuromutilin susceptibility.

Sequence type	No. of isolates	A443G^a^	Asn148Ser^a^	Pleuromutilin susceptibility		
				S	I	R
**ST112**	8	-	-	4	0	4
**ST8**	4	+	+	3	0	1
	3	-	-	3	0	0
**ST52**	2	+	+	1	1	0
	14	-	-	0	1	13

S = susceptible, I = intermediate, R = resistant; SNP/amino acid substitution in the L3 gene present (+) / absent (-);

^a^ L3 mutations numbered according to the nucleotide/amino acid sequence of *B*. *pilosicoli* strain P43/6/78 (GenBank accession no. AF114845 and AAG27264, respectively) [16].

### Distribution of virulence-associated genes (VAGs) among *B*. *hyodysenteriae* isolates

Most of the 15 VAGs tested by PCR were almost regularly present in the 116 *B*. *hyodysenteriae* field isolates. The iron-transporter genes *ftnA* and *bitC* were detected in 100% and 95.7% of the isolates, respectively. OMP gene *bhlp16* was present in 70 isolates (60.3%), while *bhlp17*.*6* and *bhmp39h* could not be amplified from any of the field isolates. *Bhlp29*.*7* was present in all isolates, whereas 87.9% of the isolates possessed *bhmp39f*. The eight hemolysin genes *tlyA*, *tlyB*, *tlyC*, *hlyA*, *BHWA1_RS04705*, *BHWA1_RS09085*, *BHWA1_RS02195*, and *BHWA1_RS02885* were regularly detected in all isolates. VAG typing of the reference strains revealed comparable results, with both strains being positive for all genes but *bhlp17*.*6* (B204) and *bhlp16* as well as *bhmp39h* (B8044). Overall, we could determine five different VAG patterns among our *B*. *hyodysenteriae* field isolates (Table 3). The most prevalent pattern (type 1) was represented by 62 isolates that each harbored 13 out of 15 VAGs. Interestingly, the presence of *bhmp39f* varied among isolates recovered from four of the six farms that had submitted samples repeatedly. In two farms (no. 31 and 81) the initial isolates did not possess the *bhmp39f* gene whereas it was present in those isolates recovered 12 and 13 months later. In the two other farms (no. 40 and 43) an exactly opposite situation was observed with initial isolates harboring *bhmp39f* and late isolates being negative for the gene (S2 Table).

**Table 3:** Patterns of virulence-associated genes (VAGs) in 116 *B*. *hyodysenteriae* field isolates from pigs in Germany.

Pattern type (no. of isolates)	VAG categories								Multilocus sequence type (ST)
	Hemolysis	Iron metabolism		Outer membrane proteins					
	hemolysin genes*	*ftnA*	*bitC*	*bhlp16*	*bhlp17*.*6*	*bhlp29*.*7*	*bhmp39f*	*bhmp39h*	
1 (n = 62)	+	+	+	+	-	+	+	-	ST8, ST52, ST112, ST113, ST114, ST115, ST117, ST118, ST120, ST123, ST124
2 (n = 36)	+	+	+	-	-	+	+	-	ST52, ST122, ST131, ST193
3 (n = 4)	+	+	-	-	-	+	+	-	ST52, ST116
4 (n = 5)	+	+	+	-	-	+	-	-	ST52, ST116
5 (n = 8)	+	+	+	+	-	+	-	-	ST8, ST52, ST112, ST114, ST121
6 (n = 1)	+	+	-	-	-	+	-	-	ST52

* *tlyA*, *tlyB*, *tlyC*, *hlyA*, *BHWA1_RS02885*, *BHWA1_RS02195*, *BHWA1_RS09085*, *BHWA1_RS04705;* + = gene present, − = gene absent

### VAGs and antimicrobial resistance in relation to sequence types of *B*. *hyodysenteriae*

Due to the overall presence (hemolysin genes, iron-transferring genes, and OMP encoding genes *bhlp29*.*7* and *bhmp39f*) or absence (*bhlp17*.*6* and *bhmp39h*) of VAGs in most of our isolates, to establish a significant link between VAGs and STs was not possible. Merely the OMP gene *bhlp16* showed a ST-related distribution ((*p*<0.0001), χ2 test), with all ST8 and ST112 isolates harboring this gene, while it was only present in 14.6% of ST52 isolates.

Regarding the three predominant STs in this study, isolates with presumed susceptibility to tiamulin and valnemulin were significantly associated with ST8 (MIC_90_ tiamulin: 1 μg/ml, valnemulin: 0.125 μg/ml), while isolates of ST52 (MIC_90_ tiamulin: 16 μg/ml, valnemulin: 4 μg/ml) and ST112 (MIC_90_ tiamulin: >16 μg/ml, valnemulin: >4 μg/ml) were interpreted as resistant. Differences between ST8 and ST52/ST112 were statistically supported with *p*-values of <0.0001 for tiamulin and <0.002 for valnemulin, respectively. All remaining susceptible isolates (MIC range for tiamulin ≤0.016–0.25 μg/ml, for valnemulin ≤0.004–0.063 μg/ml) were allocated to sequence types ST113, ST115, ST116, ST117, ST122, ST123, ST131, and ST193 with, however, a maximum of only three isolates per ST which did not allow for a statistical analysis. In contrast, isolates with reduced susceptibility to tiamulin (MIC range 0.031 –>16 μg/ml) and valnemulin (MIC range ≤0.004 –>4 μg/ml) belonged to other, rather underrepresented STs such as ST114, ST118, ST120, and ST121. Regarding antibiotic susceptibility over time (Table 4) a significant decrease was evident in isolates belonging to ST52 for both pleuromutilins (for tiamulin *p* = 0.011, for valnemulin *p* = 0.008). Interestingly, isolates belonging to ST112 showed a trend towards increased antimicrobial susceptibility.

**Table 4:** MIC_50_* values of pleuromutilins for *B*. *hyodysenteriae* isolates of major multilocus sequence types according to the year of isolation.

Sequence type (no. of isolates; isolates per isolation period)	Antimicrobial substance	MIC_50_ values (μg/ml)	
		Year of isolation	
		1990–2004	2005–2016
ST8 (n = 14; 11/3)	Tiamulin	0.063	0.063
	Valnemulin	0.063	0.016
ST52 (n = 48; 19/29)	Tiamulin**	2	16
	Valnemulin**	0.125	4
ST112 (n = 30; 16/14)	Tiamulin	4	2
	Valnemulin	2	0.5

* MIC: minimum inhibitory concentration (μg/ml), MIC_50_: MIC at which 50% of the isolates are inhibited in growth;

**significant rate (*p*<0.05) of the comparison between 1990–2004 and 2005–2016.

Comprehensive data of the isolates including the year of isolation, farm of origin, antimicrobial susceptibility, and sequence type are provided in S2 Table.

### Determination of a possible linkage between the sequence type of *B*. *hyodysenteriae* and hemolysin gene sequences

Based on their nucleotide sequences (NS) four of the seven genes, namely the putative hemolysin genes *BHWA1_RS09085*, *BHWA1_RS02885*, *BHWA1_RS02195* (*yplQ*) as well as *tlyB*, showed a significant (*p*<0.05) correlation towards the concatenated NS of the seven housekeeping genes used for MLST analysis when tested with the Mantel test. When comparing the amino acid (AA) sequences of these hemolysins, only BHWA1_RS02195 (YplQ) showed a significant (*p*<0.0013) linkage with the concatenated AA sequences of the housekeeping genes of different STs. In contrast, the hemolysin genes *BHWA1_RS09085*, *BHWA1_RS02885*, *tlyA*, *tlyC*, and *hlyA* did not correlate with the concatenated sequences (NS and AA) of the housekeeping genes (*p*> 0.05).

The neighbor-joining trees of the hemolysin genes *BHWA1_RS02885* and *BHWA1_RS02195* compared to the concatenated NS of the seven housekeeping genes are provided as examples in S2 and S3 Figs.

## Discussion

This study determined the clonal diversity of *B*. *hyodysenteriae* isolates from different regions of Germany and explored possible links between the phylogenetic background and the susceptibility of these isolates to pleuromutilins as well as to the presence of virulence gene profiles compiled from data of 15 VAGs.

We observed a predominance of ST8, ST52, and ST112 among *B*. *hyodysenteriae* isolates in Germany. Of the 421 *B*. *hyodysenteriae* isolates currently listed on the MLST website (http://pubmlst.org/brachyspira/) (accessed at 09/08/2017) only one of the 196 non-European isolates has been assigned to one of these STs. In contrast, all three STs were frequently present among isolates circulating in different European countries, such as Italy, Spain, Belgium, and Poland. Hence, ST8, ST52, and ST112 seem to be geographically restricted, with the only exception of one ST52 isolate that was submitted from Japan (data from this study). The emergence of “European” *B*. *hyodysenteriae* STs may result from the enormous pig trade movement among European countries. This has recently been discussed as a probable reason for the wide dissemination of antimicrobial resistant *B*. *hyodysenteriae* isolates in Europe [14, 15]. On the other hand, trade restrictions may lead to uneven geographical developments. Australia, for example, has banned the import of pigs in the mid-1980s [41]. This reasonably explains why STs observed among Australian *B*. *hyodysenteriae* isolates differ from those of other countries (Fig 1A). Similarly, of isolates of *B*. *hampsonii* clade II and I only those from clade I (KC35 and EB106, GenBank accession no. JX197410 and JX197409) have been isolated in Europe [6, 7].

The distribution of most of the German isolates amongst only a few STs (ST8, ST52, and ST112) is a finding that has been observed in other countries as well. In Italy (ST77, ST79 and ST83), the United States (ST93), and Spain (ST8 and ST71), certain STs were predominant and this suggests a local expansion of distinct clonal groups. On the other hand, there are several indications for the diversification of the pathogen, such as the presence of several single allele variants of ST52, the finding of numerous other STs among German *B*. *hyodysenteriae* isolates, the identification of genetically different isolates in the United States over four decades [13], and the development of different STs or MLVA (multilocus variable number tandem repeat analysis) types within the same CC over time in Italy [14]. In recent studies from Italy and Australia [15, 41] different STs in the same farm were observed, but most of them represented single locus variants that belonged to the same clonal complex, suggesting a high relatedness between the isolates.

In our study isolates originating from the same farm showed the same ST over longer time periods, such as ST120, that has been isolated on farm no. 95 in an interval of 19 months. This finding might indicate a long-term persistence of certain *B*. *hyodysenteriae* genotypes in pig farms. Deeper clonal analyses of such isolates would be necessary to determine, if even identical strains do persist over a certain time on pig farms. Re-entry of isolates through purchase of pigs from other farms and/or countries or through other sources such as mice, which have been suggested as a potential reservoir of *B*. *hyodysenteriae* [13], would also give reasonable explanations for the maintenance of STs in pig farms.

Although we included all published or submitted *B*. *hyodysenteriae* isolates for which STs have been published and/or submitted to the MLST website in our MST, the current picture of the population structure of *B*. *hyodysenteriae* as well as that for the temporal distribution of STs is likely biased by different study designs and by the lack of data from certain countries with relevant pig populations, such as The Netherlands, Norway, France, and several non-European countries. The availability of a representative MLST dataset of global *B*. *hyodysenteriae* isolates would be of immense value to identify possible transmission routes between pig herds on a national and international level [2, 10, 11, 12, 15]. Also the MST depicting the temporal distribution of STs (Fig 1B) must be interpreted with caution as it can only consider strains for which MLST and whole genome sequence data are available. Thus, it cannot be determined whether distinct STs such as ST112, ST83, ST77, ST76, ST93, and ST94 are indeed genotypes that have evolved most recently. To this time, it seems the there is an ongoing diversification of the pathogen *B*. *hyodysenteriae*. This has also been hypothesized in a recent study, where only six of 69 STs from *B*. *hyodysenteriae* isolates from Australia were maintained over time (1980–2000 until 2014–2016). This may either indicate an ongoing evolution of the pathogen or a source of isolates with highly diverse genotypes [41]. When combining the data of both MSTs (Fig 1A and 1B) presented in our study, ST8 and ST52 seem to play a key role in European countries. The first time of isolation for both STs was in the 1990s with both being continuously detected in the 2000s and the 2010s.

Regarding the VAGs of *B*. *hyodysenteriae*, this is one of the first studies that analyzed a representative number of isolates for all known and putative hemolysin genes. The almost regular detection of the eight hemolysin genes in our isolates confirms data from whole genome sequencing, where *B*. *hyodysenteriae* isolates also contained all these genes [3, 31]. Besides the fact that the hemolysin genes seem to be ubiquitous, they have a strikingly high homology rate independent of the ST of the isolate. In our study, the hemolysin genes *BHWA1_RS02195* and *BHWA1_RS02885* were those with the highest variety in the nucleotide sequences (identity 97.8% and 99.0%, respectively). This is in accordance with previously published findings, where both genes showed the highest amount of variety [3, 24]. By comparing the nucleotide sequences of hemolysin genes with the concatenated housekeeping genes, largely reflecting the core genome of the isolates, we observed that the putative hemolysin genes most likely followed the phylogeny of the core genome. On the AA level this correlation was only seen for one of them, namely BHWA1_RS02195 (YplQ), indicating a higher presence of non-synonymous mutations. Other hemolysin genes, such as *tlyA*, *tlyC*, and *hlyA* seem to be less conserved than the putative genes and thus might have undergone an independent evolution. This is likely controlled by other factors, which are unknown so far, than those mediating the phylogeny. Although this is rather speculative, it seems to be a most reasonable explanation for these observations.

Of the other VAGs the OMP encoding *bhlp29*.*7* was present in all 116 isolates. This is in accordance with a similar study demonstrating the gene in all (n = 23) examined isolates [3]. Due to the wide distribution of this gene, whose function has not finally been resolved [33], it sounds convincing that Bhlp29.7 was previously recommended as suitable antigen for the serological diagnosis of SD [42]. Of the *bhmp39a-h*, forming a set of paralogous genes, *bhmp39f* was present in almost all isolates, while *bhmp39h* could not be detected at all. This is in contrast to former studies that demonstrated a wide distribution of both genes [43]. The remaining OMPs encoding genes (*bhlp16* and *bhlp17*.*6*) are suggested to be located on the same gene locus, so that one isolate either harbors one or the other gene [44]. Interestingly, 46 of our isolates neither contained *bhlp16* nor *bhlp17*.*6* which has also been reported in other studies [3, 32]. The absence of both genes was verified by use of additional primers (S1 Table) in the flanking region of the genes. Considering the sequence type, it is notable that only a few isolates (7 of 52) of the CC52 (i.e. ST52, ST116, ST131, and ST193 of this study) harbored the *bhlp16* gene. Conclusive studies about the role of *bhlp16* are not available so far, but since this gene is assumed to play a role in immune evasion [45] genetically different *B*. *hyodysenteriae* isolates must operate with different OMPs or pathways. Interestingly, also the occurrence of *bhmp39f* varied among isolates originating from the same farm. A reasonable explanation implicates that *bhmp39f* is not essential for the viability of the pathogen as it has recently been concluded for some plasmid genes possibly associated with virulence [41]. It should also be emphasized, that any negative finding could be the result of a false negative PCR due to variations in primer binding sites, which may particularly be the case in OMP genes that are involved in antigenic variability [43]. Whether mismatches result in an altered functionality of the protein cannot be determined at this moment.

Genes encoding iron-transfer proteins were also confirmed to be widely present in *B*. *hyodysenteriae*, which might be due to their essential role for the bacteria’s vitality and virulence. Accordingly, the Bit system is absent in apathogenic *B*. *innocens* or less virulent *B*. *pilosicoli* [46]. In summary, most of the investigated VAGs, except for *bhlp16*, were widespread among *B*. *hyodysenteriae* isolates, which made it impossible to determine any linkages between the sequence type and possession of distinct virulence genes. However, we could demonstrate that isolates belonging to the same ST did not necessarily share the same virulence gene pattern. For example, the predominant ST52 contained all six virulence patterns found in this study. Nevertheless, it must be considered that PCR has a lower sensitivity than whole genome sequencing (WGS). Recently published data about WGS could show an accordance of STs with VAGs [3].

Data of antimicrobial susceptibility testing (40.9% pleuromutilin resistant isolates) confirmed the trend towards a reduced antimicrobial susceptibility of *B*. *hyodysenteriae* isolates from Germany to tiamulin and valnemulin in recent years [29, 47]. Pleuromutilin resistance of *B*. *hyodysenteriae* is not limited to German isolates but has been observed in other countries as well. A recent study from Japan revealed high MIC_90_-values of tiamulin (6,25 μg/ml) and valnemulin (6,25 μg/ml) of 29 *B*. *hyodysenteriae*-isolates [48]. Also in Spain and the Czech Republic trends towards a reduced antimicrobial susceptibility of *B*. *hyodysenteriae* isolates were reported [49, 50]. In contrast, Kirchgässner et al. (2016) [51] reported full susceptibility of *B*. *hyodysenteriae* isolates from Switzerland towards pleuromutilins, which might be due to limited trade activities of this non-EU country with the EU countries. Since the first detection of *B*. *hyodysenteriae* in fattening pig farms was in 2008 and in multiplier herds in 2016 [52] it can be assumed that selected therapy was rather uncommon, which leads to a low selection pressure. Our data indicate a correlation between the ST and pleuromutilin resistance of *B*. *hyodysenteriae*. Resistant isolates were predominantly found among ST52 and ST112, and isolates of ST52 with reduced susceptibility to pleuromutilins very likely emerged during the past 25 years (MIC_50_ for valnemulin increased from 0,125 μg/ml to 4 μg/ml) (Table 4). In contrast, isolates belonging to ST112 showed decreased MIC_50_-values for both pleuromutilins throughout the study period with a change from resistant to intermediate MIC_50_ values. However, these findings do not necessarily reflect a general trend in resistance of *B*. *hyodysenteriae* to pleuromutilins since these data only refer to STs that contained a statistically representative number of isolates. If the observed differences in antimicrobial susceptibility may be due to increased hygienic efforts and infection control programs as well as an appropriate feeding and management, probably accompanied by a reduced usage of antibiotics, as recently reported, remains elusive [1, 53]. Furthermore, mutations in ribosomal proteins can lead to increased susceptibility of *B*. *hyodysenteriae* isolates to pleuromutilins, like it was already demonstrated for the ribosomal protein L3 [17]. However, in two of six pig farms (no. 43 and 95) isolates recovered in intervals of three weeks and 19 months, respectively, changed in their antimicrobial susceptibility from susceptible to resistant. A fast shift towards resistance, as it was the case in farm no. 43, probably indicates the presence of already resistant strains in the pig population. Wide use of antibiotics in pig production including inappropriate dosages, incorrect application intervals and poor housing conditions as well as entry of resistant isolates by animal trade are plausible reasons for the increase of antimicrobial resistance [1, 54, 55].

Analyses of the ribosomal protein L3 encoding gene among isolates of the predominant STs, however, were not conclusive, except that the relevant SNP occurred more often in ST8 than in ST52 or ST112 isolates. The fact that changes in antimicrobial susceptibility over time caused by mutations in ribosomal genes coincide with unchanged STs, is very likely explainable by differences in the location and mutation rates of the genes that predict resistance and ST, respectively.

The proposed CC3 of Rugna et al. (2015), which resembles CC52 in our study, contains several STs, such as ST52, ST116, ST131, and ST193 that comprise a large number of pleuromutilin resistant isolates, with only marginal differences between countries [15]. Referring to another study from Italy, CC4 isolates (CC8 in our study) were largely susceptible to valnemulin [14], which was also true for our ST8 (CC8) isolates. This is also in accordance with a recent study from Belgium where ST8 isolates were mostly susceptible to pleuromutilins [56].

## Conclusion

Our study revealed the presence of 15 STs of *B*. *hyodysenteriae* in Germany with three of them being dominant (ST8, ST52, and ST112). They are present in several European countries which may be due to the vast trading activities with pigs within the EU. More than one third of our isolates were resistant to both pleuromutilins that currently represent the most effective antibiotic substances used for the control of SD. Pleuromutilin resistance as well as susceptibility could be linked with CCs or even STs, suggesting a predictive role of MLST for the resistance phenotype of an isolate.

Regarding VAGs, only the OMP gene *bhlp16* was negatively correlated with a certain group of isolates (CC52). All other VAGs including the hemolysin genes were either regular present or completely absent among the investigated isolates and a linkage to their STs could not be determined. Comparing nucleotide and amino acid sequences, we could show that the putative hemolysin genes most likely followed the phylogeny of the core genome whereas the other hemolysin genes, such as *tlyA*, *tlyC*, and *hlyA*, were less conserved and might have undergone an independent evolution, most probably due to other kinds of selective pressure. Extending the set of global, well-characterized isolates would be very helpful in broadening our understanding of the molecular epidemiology of *B*. *hyodysenteriae* isolates. Comprehensive MLST and virulence data could help to select epidemiologically relevant isolates for further analysis, such as whole genome sequencing, transcriptome analysis or targeted pathogenicity studies, and finally for the selection of vaccine candidates.

## Supporting information

S1 Fig BURST algorithm based on 746 global *B*. *hyodysenteriae* isolates. (DOCX)

S2 Fig Neighbour-Joining Tree (NJT) of hemolysin gene *BHWA1_RS02195* (*yplQ*) and the concatenated nucleotide sequences of the housekeeping genes used for MLST. (DOCX)

S3 Fig Neighbour-Joining Tree (NJT) of hemolysin gene *BHWA1_RS02885* (*hly*) and the concatenated nucleotide sequences of the housekeeping genes used for MLST. (DOCX)

S1 Table Primers and PCR conditions used in this study. (DOCX)

S2 Table Characteristics of 116 *B*. *hyodysenteriae* isolates from pigs in Germany: Sequence type, year of isolation, farm of origin, antimicrobial susceptibility and mutation of the ribosomal protein L3. (DOCX)
