# Supplementary material for: Phylogenetic diversity, antimicrobial susceptibility and virulence gene profiles of Brachyspira hyodysenteriae isolates from pigs in Germany
Source: PLoS One. 2018 Jan 11;13(1):e0190928. doi: 10.1371/journal.pone.0190928 (PMC5764319; doi:10.1371/journal.pone.0190928)

**S1 Fig. BURST algorithm based on 746 global *B. hyodysenteriae* isolates.** The labelled dots represent the sequence type (ST), the size of the dot depicts the number of isolates represented by that ST and solid lines between two dots indicate that they are single locus variants (SLV) of each other and belong to a clonal complex (CC). Blue dots represent the potential founder of the CC.


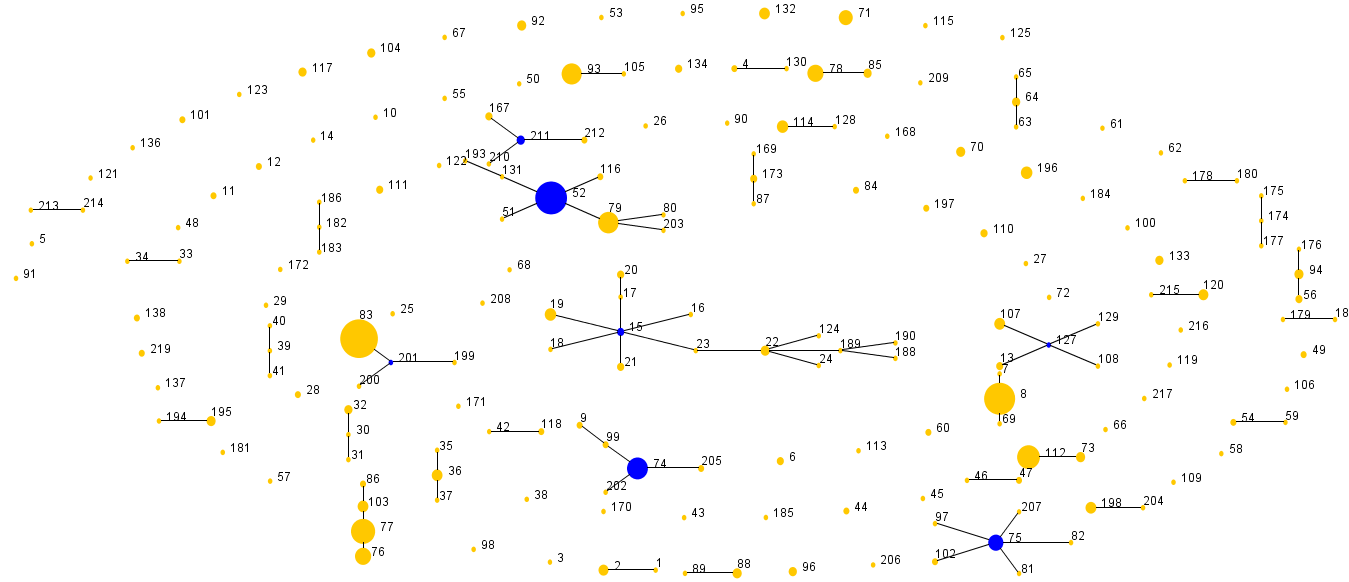

Supplement: S1 Fig — (DOCX) [file pone.0190928.s001.docx]
