# Supplementary material for: Phylogenetic diversity, antimicrobial susceptibility and virulence gene profiles of Brachyspira hyodysenteriae isolates from pigs in Germany
Source: PLoS One. 2018 Jan 11;13(1):e0190928. doi: 10.1371/journal.pone.0190928 (PMC5764319; doi:10.1371/journal.pone.0190928)

**S3 Fig. Neighbour-Joining Tree (NJT) of hemolysin gene *BHWA1_RS02885* (*hly*) and the concatenated nucleotide sequences of the housekeeping genes used for MLST.** NJT A represents the concatenated nucleotide sequences of the housekeeping genes used for MLST, while NJT B is representing the nucleotide sequence of hemolysin gene *BHWA1_RS02885* (*hly*) of 29 isolates. The potential coevolution between housekeeping genes and hemolysin genes was determined with the Mantel test. The *p*-value for *hly* was >0.05.


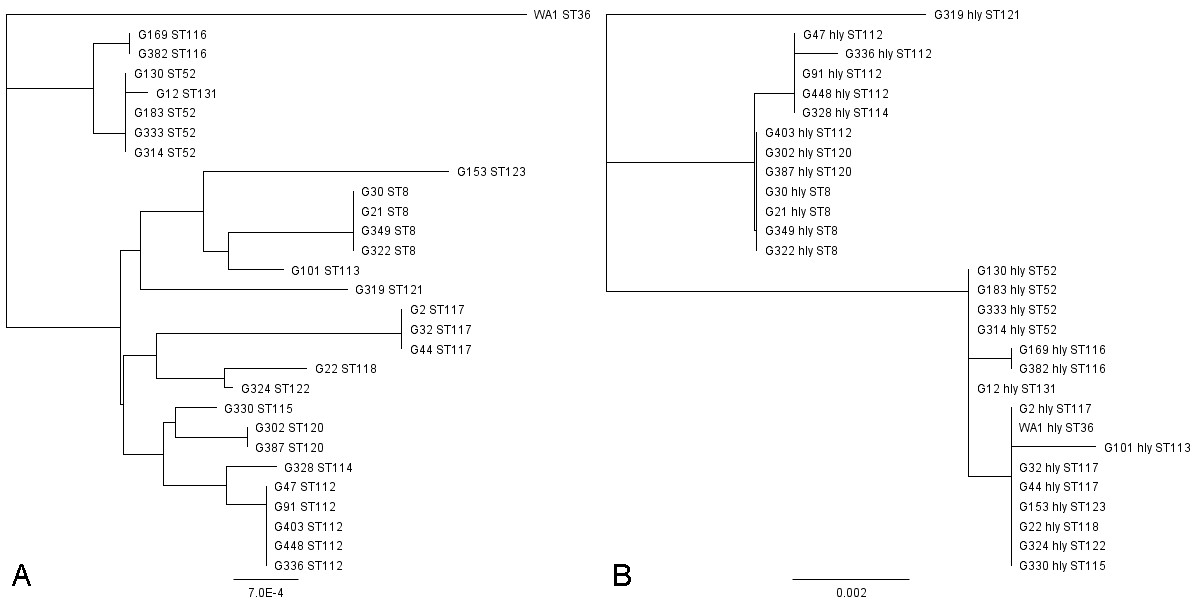

Supplement: S3 Fig — (DOCX) [file pone.0190928.s003.docx]
