# Supplementary material for: Phylogenetic diversity, antimicrobial susceptibility and virulence gene profiles of Brachyspira hyodysenteriae isolates from pigs in Germany
Source: PLoS One. 2018 Jan 11;13(1):e0190928. doi: 10.1371/journal.pone.0190928 (PMC5764319; doi:10.1371/journal.pone.0190928)
Supplement: S1 Table — (DOCX) [file pone.0190928.s004.docx]

**S1 Table. Primers and PCR conditions used in this study.**

| Primer | Target gene | Nucleotide sequence (5' - 3') | Amplicon size [bp] | Annealing | | Reference strain | Reference |
| --- | --- | --- | --- | --- | --- | --- | --- |
|  |  |  |  | [°C] | [s] |  |  |
| ftnF | *ftnA* | AGCTTGAAAGGATGCAGCAGC | 292 | 54 | 60 | B204 | Davis et al. (1) |
| ftnR |  | CTTCTTCTTCAAGCTGTTC |  |  |  |  |  |
| BitC-2526 | *bitC* | GATATACTTTGGGGCGGAACTA | 265 | 54 | 60 | B204 | Hue (2) |
| BitC-2790 |  | AAGAAGATGAAGAGGCAGAAGGAT |  |  |  |  |  |
| BhtlyAF | *tlyA* | GCAGATCTAAAGCACAGGAT | 527 | 60 | 40 | B204 | Råsbäck et al. (3) |
| BhtlyAR |  | GCCTTTTGAAACATCACCTC |  |  |  |  |  |
| TlyBfor | *tlyB* | TGAAGAGGGAGGCGGACTTA | 613 | 57 | 40 | B204 | This study |
| TlyBrev |  | AGAGCCATCATTAGCATCAACG |  |  |  |  |  |
| TlyCfor | *tlyC* | GGTTGTTGATGAATACGGCGG | 252 | 60 | 40 | B204 | This study |
| TlyCrev |  | GGAAGCCTGCCCAAGTATGA |  |  |  |  |  |
| BHlyAneu-for | *hlyA* | TCACTGATACAGCTTCTTTCG | 148 | 60 | 40 | B204 | This study |
| BHlyAneu-rev |  | GTATTTAGCAGCATCAGCTAC |  |  |  |  |  |
| hlyneu-for | *BHWA1_RS02885* | CGCCYCGTGTTGATATGGTA | 177 | 57 | 40 | B204 | This study |
| hlyneu-rev |  | TCTTTCCGCCGCCTTTAACA |  |  |  |  |  |
| YplQfor | *BHWA1_RS02195* | ACAGGAGTATATGTTGCGGCT | 223 | 60 | 40 | B204 | This study |
| YplQrev |  | CCCATGGCATAGGAGCTTTACT |  |  |  |  |  |
| 1870for | *BHWA1_RS09085* | CCACGCCATCTGTATCCGAA | 322 | 60 | 40 | B204 | This study |
| 1870rev |  | CGGCAATAGCTGTCTTAGTGC |  |  |  |  |  |
| 962for | *BHWA1_RS04705* | AACTTCTCCCCCTTGTCTGC | 355 | 60 | 40 | B204 | This study |
| 962rev |  | CTCAGGCGATACAGTTGCCA |  |  |  |  |  |
| smp-f | *bhlp16* & *17.6* | GTTCTTAAATAATACCATAATC | 768 | 62 | 60 | B204, | Barth et al. (4) |
| smp-r |  | CCGCCAGTCAAATAATCTTTTAAT |  |  |  | B8044 |  |
| SmpA forward | *bhlp 16* | AAATGAACAAAAAAATTTTCACAC | 509 | 62 | 60 | B204 | Barth et al. (4) |
| SmpA reverse |  | AGCGCTAGCTCCCCAATTTTCTTTAG |  |  |  |  |  |
| SmpB1 | *bhlp 17.6* | TTGAAGTAAAAGCTCAAGAT | 375 | 55 | 60 | B8044 | Barth et al. (4) |
| SmpB2 |  | TGTACTAGCTGGTGTAAGTC |  |  |  |  |  |
| BmpB58 | *bhlp 29.7* | TGCGGAAATACTTCTTCTGGTG | 527 | 55 | 30 | B204 | La et al. (5) |
| BmpBR630 |  | AGAACCAGGATTCAAACCGAAG |  |  |  |  |  |
| *Vsp_for* | *bhmp39f* | ACTATTAAAGGTACTTTCGG | 437 | 55 | 30 | B204 | This study |
| *Vsp_F* |  | ATACTCACTAGCTCCAGCTC |  |  |  |  |  |
| *bhmp39-for* | *bhmp39h* | ATGAAAAAAGTTTTATTGACAGCTA | 1170 | 68 | 60 | B204 | This study |
| *bhmp39h-rev* |  | TTGAGCACCGCCTAAAGCAGGTAA |  |  |  |  |  |
| Bra1 | *nox* | GCTAGTCCTGAAAGTTTGAGAGG | 435 | 60 | 30 | B204 | Herbst et al. (6) |
| Bra2 |  | AGCTTCATCAGTGATTTCTTTATCA |  |  |  |  |  |

**References:**

1. Davis AJ, Smith SC, Moore RJ. The *Brachyspira hyodysenteriae ftnA* gene: DNA vaccination and real-time PCR quantification of bacteria in a mouse model of disease. Curr Microbiol. 2005;50(6):285–91. doi: 10.1007/s00284-005-4472-2. PMID: 15968507.
2. Hue M. Etablierung einer Polymerasekettenreaktion zum Nachweis von *Brachyspira hyodysenteriae* aus Schweinekot. Doctoral dissertation, the University of Veterinary Medicine Hannover; 2005. Availale from: <http://elib.tiho-hannover.de/dissertations/huem_ss05.pdf>
3. Råsbäck T, Fellström C, Gunnarsson A, Aspán A. Comparison of culture and biochemical tests with PCR for detection of *Brachyspira hyodysenteriae* and *Brachyspira pilosicoli*. J Microbiol Methods. 2006;66(2):347–53. doi: 10.1016/j.mimet.2005.12.008. PMID: 16457900.
4. Barth S, Gömmel M, Baljer G, Herbst W. Demonstration of genes encoding virulence and virulence life-style factors in *Brachyspira* spp. isolates from pigs. Vet Microbiol. 2012;155(2-4):438–43. doi: 10.1016/j.vetmic.2011.09.032. PMID: 22047713.
5. La T, Tan P, Phillips ND, Hampson DJ. The distribution of *bmpB*, a gene encoding a 29.7kDa lipoprotein with homology to MetQ, in *Brachyspira hyodysenteriae* and related species. Vet Microbiol. 2005;107(3-4):249–56. doi: 10.1016/j.vetmic.2005.01.016.
6. Herbst W, Willems H, Baljer G. Verbreitung von *Brachyspira hyodysenteriae* und *Lawsonia intracellularis* bei gesunden und durchfallkranken Schweinen. Berl Munch Tierarztl Wochenschr. 2004;117(11-12):493–8. PMID: 15584431.
