# Supplementary material for: Phylogenetic diversity, antimicrobial susceptibility and virulence gene profiles of Brachyspira hyodysenteriae isolates from pigs in Germany
Source: PLoS One. 2018 Jan 11;13(1):e0190928. doi: 10.1371/journal.pone.0190928 (PMC5764319; doi:10.1371/journal.pone.0190928)
Supplement: S2 Table — (DOCX) [file pone.0190928.s005.docx]

**S2 Table. Characteristics of 116 *B. hyodysenteriae* isolates from pigs in Germany: sequence type, year of isolation, farm of origin, antimicrobial susceptibility and mutation of the ribosomal protein L3.**

| **Isolate name** | **Sequence type** | **Year of isolation** | **Farm of origin** | **Tiamulin MIC (µg/ml)** | **Valnemulin MIC (µg/ml)** | **Amino acid substitution Asn148Ser** |
| --- | --- | --- | --- | --- | --- | --- |
| G327 | 8 | 1990 | 1 | 0.063 | 0.063 | + |
| G325 | 8 | 1991 | 2 | 0.063 | 0.063 | - |
| G326 | 8 | 1991 | 3 | 0.063 | 0.031 | ND |
| G322 | 8 | 1994 | 4 | 0.063 | 0.063 | - |
| G323 | 8 | 1994 | 5 | 0.063 | 0.063 | + |
| G321 | 8 | 1997 | 6 | 0.125 | 0.063 | + |
| G318 | 8 | 2000 | 7 | 8 | 1 | + |
| G048 | 8 | 2003 | 8 | 0.031 | ≤0.004 | ND |
| G021 | 8 | 2004 | 9 | 0.125 | ≤0.004 | + |
| G030 | 8 | 2004 | 10 | 0.063 | ≤0.004 | ND |
| G061 | 8 | 2004 | 11 | 0.031 | ≤0.004 | - |
| G134 | 8 | 2006 | 12 | 0.063 | ≤0.004 | ND |
| G141 | 8 | 2006 | 13 | 0.063 | 0.016 | ND |
| G349 | 8 | 2011 | 14 | 1 | 0.125 | ND |
| G333 | 52 | 1998 | 15 | 4 | 4 | - |
| G316 | 52 | 1999 | 16 | 1 | 1 | - |
| G317 | 52 | 1999 | 17 | 8 | 2 | - |
| G332 | 52 | 2000 | 18 | 2 | 4 | - |
| G314 | 52 | 2001 | 19 | 4 | 2 | - |
| G315 | 52 | 2001 | 20 | 4 | 0.125 | + |
| G307 | 52 | 2002 | 21 | 8 | 2 | ND |
| G334 | 52 | 2002 | 22 | 8 | 2 | - |
| G337 | 52 | 2002 | 23 | 8 | 4 | - |
| G338 | 52 | 2002 | 24 | 8 | 2 | - |
| G007 | 52 | 2003 | 25 | 0.031 | ≤0.004 | ND |
| G088 | 52 | 2003 | 26 | 0.125 | ≤0.004 | ND |
| G098 | 52 | 2003 | 27 | 2 | 0.063 | ND |
| G016 | 52 | 2004 | 28 | 0.063 | ≤0.004 | ND |
| G042 | 52 | 2004 | 29 | 0.063 | ≤0.004 | ND |
| G046 | 52 | 2004 | 30 | 2 | 0.063 | ND |
| G049 | 52 | 2004 | 31 | 0.063 | 0.008 | ND |
| G110 | 52 | 2004 | 31 | 0.063 | ≤0.004 | ND |
| G043 | 52 | 2005 | 31 | 0.125 | ≤0.004 | ND |
| G050 | 52 | 2004 | 32 | 1 | 0.063 | ND |
| G057 | 52 | 2005 | 33 | 0.125 | ≤0.004 | ND |
| G076 | 52 | 2005 | 34 | 0.125 | ≤0.004 | ND |
| G130 | 52 | 2006 | 35 | 16 | 4 | ND |
| G136 | 52 | 2007 | 36 | 0.25 | 0.016 | ND |
| G147 | 52 | 2007 | 37 | 4 | 4 | ND |
| G155 | 52 | 2007 | 38 | 0.25 | 0.008 | ND |
| G172 | 52 | 2008 | 39 | 16 | 4 | ND |
| G181 | 52 | 2009 | 40 | 2 | 2 | ND |
| G182 | 52 | 2009 | 40 | 2 | 1 | ND |
| G183 | 52 | 2009 | 40 | 4 | 2 | ND |
| G184 | 52 | 2009 | 40 | 4 | 2 | - |
| G201 | 52 | 2009 | 40 | >16 | >4 | ND |
| G202 | 52 | 2009 | 40 | >16 | >4 | - |
| G203 | 52 | 2009 | 40 | >16 | >4 | ND |
| G207 | 52 | 2009 | 40 | >16 | >4 | ND |
| G277 | 52 | 2010 | 41 | 16 | >4 | ND |
| G329 | 52 | 2010 | 42 | >16 | >4 | - |
| G339 | 52 | 2010 | 43 | 0.25 | 0.008 | + |
| G367 | 52 | 2010 | 43 | >16 | >4 | ND |
| G347 | 52 | 2011 | 44 | 0.25 | 0.25 | ND |
| G376 | 52 | 2011 | 40 | >16 | >4 | - |
| G377 | 52 | 2011 | 40 | 16 | 4 | - |
| G379 | 52 | 2011 | 40 | 16 | >4 | - |
| G390 | 52 | 2013 | 45 | >16 | >4 | ND |
| G397 | 52 | 2013 | 46 | 1 | 0.5 | ND |
| G433 | 52 | 2014 | 47 | 16 | 4 | ND |
| G465 | 52 | 2015 | 48 | 0,5 | 0.25 | ND |
| G487 | 52 | 2015 | 49 | >16 | 4 | ND |
| G003 | 112 | 2004 | 50 | 0.063 | 0.063 | ND |
| G062 | 112 | 2004 | 50 | ≤0.016 | 0.004 | ND |
| G320 | 112 | 2000 | 51 | 2 | 4 | - |
| G331 | 112 | 2000 | 52 | 4 | 2 | - |
| G306 | 112 | 2002 | 53 | 4 | 2 | ND |
| G308 | 112 | 2002 | 54 | 8 | 2 | ND |
| G309 | 112 | 2002 | 55 | 8 | 4 | ND |
| G310 | 112 | 2002 | 56 | 8 | 4 | ND |
| G311 | 112 | 2002 | 57 | 4 | 0.5 | ND |
| G312 | 112 | 2002 | 58 | 8 | 2 | ND |
| G335 | 112 | 2002 | 59 | 8 | 2 | - |
| G336 | 112 | 2002 | 60 | 8 | 2 | - |
| G015 | 112 | 2004 | 61 | 0.25 | 0.031 | - |
| G024 | 112 | 2004 | 62 | 0.25 | 0.125 | - |
| G047 | 112 | 2004 | 63 | 2 | 0.063 | ND |
| G064 | 112 | 2004 | 64 | 0.063 | 0.004 | ND |
| G017 | 112 | 2005 | 65 | 0.031 | ≤0.004 | - |
| G036 | 112 | 2005 | 66 | 0.125 | 0.063 | - |
| G038 | 112 | 2005 | 67 | 2 | 0.063 | ND |
| G078 | 112 | 2005 | 68 | 1 | 0.031 | ND |
| G091 | 112 | 2006 | 69 | 4 | 0.5 | ND |
| G139 | 112 | 2007 | 70 | >16 | >4 | ND |
| G274 | 112 | 2010 | 71 | >16 | >4 | ND |
| G296 | 112 | 2011 | 72 | 1 | 2 | ND |
| G381 | 112 | 2013 | 73 | 16 | 4 | ND |
| G385 | 112 | 2013 | 74 | 1 | 0.5 | ND |
| G403 | 112 | 2013 | 75 | 16 | 4 | ND |
| G420 | 112 | 2014 | 76 | 8 | 4 | ND |
| G448 | 112 | 2015 | 77 | 0.125 | 0.016 | ND |
| G449 | 112 | 2015 | 78 | >16 | >4 | ND |
| G101 | 113 | 2004 | 79 | 0.125 | 0.004 | - |
| G328 | 114 | 1990 | 80 | 0.063 | 0.063 | - |
| G440 | 114 | 2014 | 81 | 16 | 4 | ND |
| G483 | 114 | 2015 | 82 | 16 | >4 | ND |
| G488 | 114 | 2015 | 83 | 16 | >4 | ND |
| G490 | 114 | 2015 | 84 | 16 | >4 | ND |
| G493 | 114 | 2015 | 81 | 8 | 4 | ND |
| G504 | 114 | 2015 | 85 | >16 | >4 | ND |
| G502 | 114 | 2016 | 86 | ND | ND | ND |
| G330 | 115 | 2010 | 87 | ≤0.016 | ≤0.004 | - |
| G169 | 116 | 2008 | 88 | 0.125 | ≤0.004 | ND |
| G382 | 116 | 2013 | 89 | 0.125 | ≤0.004 | ND |
| G002 | 117 | 2004 | 90 | 0.063 | 0.004 | ND |
| G032 | 117 | 2004 | 91 | ≤0.016 | 0.004 | + |
| G044 | 117 | 2004 | 92 | 0.031 | 0.004 | ND |
| G022 | 118 | 2004 | 93 | 4 | 0.063 | ND |
| G302 | 120 | 2012 | 94 | 2 | 2 | ND |
| G387 | 120 | 2013 | 95 | 0.031 | ≤0.004 | ND |
| G485 | 120 | 2015 | 95 | 8 | >4 | ND |
| G319 | 121 | 2000 | 96 | 8 | 4 | - |
| G324 | 122 | 1992 | 97 | 0.125 | 0.063 | - |
| G153 | 123 | 2007 | 98 | 0.031 | ≤0.004 | ND |
| G012 | 131 | 2004 | 99 | 0.031 | 0.004 | + |
| G009 | 193 | 2005 | 100 | 0.25 | ≤0.004 | ND |

ND = not determined; + = mutation at amino acid position 148; - = no mutation at amino acid position 148
